# Supplementary material for: Association Mapping across Numerous Traits Reveals Patterns of Functional Variation in Maize
Source: PLoS Genet. 2014 Dec 4;10(12):e1004845. doi: 10.1371/journal.pgen.1004845 (PMC4256217; doi:10.1371/journal.pgen.1004845)
Supplement: Table S1 — Category counts. (DOCX) [file pgen.1004845.s004.docx]

**Table S1: Category counts**

| **Category** | **Input counts** | **GWAS counts** |  | **Permutation counts^ab^** | **Enrichment^a^** | **p-value^a^** |
| --- | --- | --- | --- | --- | --- | --- |
| Gene-proximal SNPs | 7,505,673 | 1,654 |  | 1,166.1 ± 75.56 | 1.42 | 1.07 x 10^-10^ |
| Genic CNVs | 35,743 | 24 |  | — | — | — |
| Intergenic SNPs | 17,570,581 | 1,720 |  | 2,714.8 ± 118.95 | 0.63 | 6.11 x 10^-17^ |
| Intronic SNPs | 2,246,611 | 542 |  | 350.5 ± 30.98 | 1.55 | 6.38 x 10^-10^ |
| Missense SNPs | 409,728 | 153 |  | 63.7 ± 9.46 | 2.4 | 3.85 x 10^-21^ |
| Other genic SNPs | 100,940 | 31 |  | 15.7 ± 4.18 | 1.98 | 2.47 x 10^-4^ |
| Synonymous SNPs | 416,452 | 164 |  | 64.9 ± 9.77 | 2.53 | 3.62 x 10^-24^ |
| UTR SNPs | 694,926 | 220 |  | 108.3 ±14.40 | 2.03 | 8.61 x 10^-15^ |
| Window-based CNVs | 768,430 | 294 |  | — | — | — |
| *total* | 29,749,084 | 4,802 |  |  |  |  |

^a^One million circular permutations of each chromosome were performed to determine empirical enrichment of each SNP category; CNVs were excluded due to ambiguity in their precise placement on chromosomes, especially for duplications. The resulting normal distribution of counts was used to extrapolate two-sided p-values for enrichment, since the actual values were generally more extreme than any observed permutation.

^b^mean ± standard deviation among all permutations
