# Supplementary material for: Association Mapping across Numerous Traits Reveals Patterns of Functional Variation in Maize
Source: PLoS Genet. 2014 Dec 4;10(12):e1004845. doi: 10.1371/journal.pgen.1004845 (PMC4256217; doi:10.1371/journal.pgen.1004845)
Supplement: Table S2 — GO term analysis. (DOCX) [file pgen.1004845.s005.docx]

**Table S2: GO term analysis**

| **GO term** | **Description** | **GWAS hits** | **Whole-genome hits** | **FDR^a^** | **Odds ratio** |
| --- | --- | --- | --- | --- | --- |
| ***Enriched terms*** | | ***(n=1,879 total terms)*** | ***(n=25,288 total terms)*** |  |  |
| *Protein kinase-related* | |  |  |  |  |
| GO:0004713 | Protein tyrosine kinase activity | 150 | 1313 | 7.58x10^-05^ | 1.66 |
| GO:0004674 | Protein serine/threonine kinase activity | 160 | 1430 | 8.92x10^-05^ | 1.62 |
| GO:0006468 | Protein amino acid phosphorylation | 167 | 1528 | 1.89x10^-04^ | 1.58 |
| GO:0004672 | Protein kinase activity | 168 | 1552 | 3.38x10^-04^ | 1.56 |
| GO:0016301 | Kinase activity | 195 | 1909 | 1.87x10^-03^ | 1.47 |
| GO:0016773 | Phosphotransferase activity, alcohol group as acceptor | 186 | 1824 | 2.59x10^-03^ | 1.46 |
| GO:0043687 | Post-translational protein modification | 186 | 1832 | 3.13x10^-03^ | 1.45 |
| GO:0043412 | Macromolecule modification | 204 | 2069 | 7.16x10^-03^ | 1.41 |
| GO:0006464 | Protein modification process | 195 | 2013 | 2.96x10^-02^ | 1.38 |
| GO:0032559 | Adenyl ribonucleotide binding | 292 | 3197 | 3.04x10^-02^ | 1.30 |
| GO:0005524 | ATP binding | 292 | 3193 | 3.04x10^-02^ | 1.30 |
| GO:0016310 | Phosphorylation | 178 | 1821 | 3.10x10^-02^ | 1.39 |
| GO:0001883 | Purine nucleoside binding | 304 | 3384 | 4.83x10^-02^ | 1.27 |
| GO:0001882 | Nucleoside binding | 304 | 3385 | 4.83x10^-02^ | 1.27 |
| GO:0030554 | Adenyl nucleotide binding | 304 | 3384 | 4.83x10^-02^ | 1.27 |
|  |  |  |  |  |  |
| *Transcription factor-related* | |  |  |  |  |
| GO:0030528 | Transcription regulator activity | 153 | 1432 | 1.87x10^-03^ | 1.53 |
| GO:0003700 | Transcription factor activity | 106 | 923 | 2.37x10^-03^ | 1.65 |
| GO:0010468 | Regulation of gene expression | 210 | 2216 | 3.81x10^-02^ | 1.34 |
| GO:0043565 | Sequence-specific DNA binding | 74 | 645 | 3.81x10^-02^ | 1.64 |
| GO:0045449 | Regulation of transcription | 205 | 2172 | 4.83x10^-02^ | 1.33 |
|  |  |  |  |  |  |
| *General regulation* | |  |  |  |  |
| GO:0009889 | Regulation of biosynthetic process | 209 | 2210 | 3.90x10^-02^ | 1.34 |
| GO:0031326 | Regulation of cellular biosynthetic process | 209 | 2210 | 3.90x10^-02^ | 1.34 |
| GO:0010556 | Regulation of macromolecule biosynthetic process | 209 | 2210 | 3.90x10^-02^ | 1.34 |
| GO:0019219 | Regulation of nucleobase, nucleoside, nucleotide and nucleic acid metabolic process | 207 | 2192 | 4.83x10^-02^ | 1.34 |
|  |  |  |  |  |  |
| *Other* |  |  |  |  |  |
| GO:0034641 | Cellular nitrogen compound metabolic process | 53 | 373 | 2.05x10^-03^ | 2.09 |
| GO:0032501 | Multicellular organismal process | 139 | 1390 | 4.83x10^-02^ | 1.41 |
|  |  |  |  |  |  |
| ***Depleted terms*** | |  |  |  |  |
| GO:0007154 | Cell communication | 14 | 1239 | 5.09x10^-22^ | 0.14 |
| GO:0030163 | Protein catabolic process | 13 | 1142 | 2.18x10^-20^ | 0.14 |
| GO:0009057 | Macromolecule catabolic process | 33 | 1391 | 5.80x10^-14^ | 0.29 |
| GO:0009056 | Catabolic process | 50 | 1552 | 9.29x10^-10^ | 0.40 |
| GO:0007165 | Signal transduction | 32 | 1184 | 1.00x10^-09^ | 0.33 |

^a^Only terms with FDR < 0.05 are shown
